# Draft Genome Sequences of Novel Sequence Type 3559 Carbapenem-Resistant Klebsiella pneumoniae Isolates Recovered from the Environment

**DOI:** 10.1128/MRA.00518-19

**Published:** 2019-06-06

**Authors:** Mutshiene Deogratias Ekwanzala, Phumudzo Budeli, John Barr Dewar, Ilunga Kamika, Maggy Ndombo Benteke Momba

**Affiliations:** a Department of Environmental, Water, and Earth Sciences, Tshwane University of Technology, Arcadia Campus, Pretoria, South Africa; b Department of Metallurgical, Chemical Engineering and Material Sciences, Tshwane University of Technology, Pretoria, South Africa; c Department of Life and Consumer Sciences, University of South Africa, Florida Campus, Johannesburg, South Africa; d Department of Environmental Sciences, University of South Africa, Science Campus, Johannesburg, South Africa; Georgia Institute of Technology

## Abstract

Here, we report a novel sequence type, 3559, from four genomes of Klebsiella pneumoniae isolates from South African hospital wastewater, influent wastewater, river water, and riverbed sediment. The genome annotation indicated a wide variety of resistance genes, including *bla*_KPC-2_, and virulence factors revealing their possible pathogenicity.

## ANNOUNCEMENT

Klebsiella pneumoniae, a member of the *Klebsiella* genus, is an established important pathogen in nosocomial infections. This Gram-negative encapsulated nonmotile bacterium can cause infections in both humans and animals. Such infections in humans include pneumonia, thrombophlebitis, urinary tract infection, cholecystitis, diarrhea, upper respiratory tract infection, wound infection, osteomyelitis, meningitis, bacteremia, and septicemia (1). Carbapenem-resistant K. pneumoniae (CRKP) infections have limited treatment options. In 2017, the World Health Organization added carbapenem-resistant Klebsiella pneumoniae as a critical pathogen for research and development of alternative antibiotics because of its severity and because pan-resistant strains are emerging (2). Hence, here, we present a novel sequence type isolated from different compartments of a wastewater treatment plant and its receiving water body.

Four CRKP isolates were recovered from hospital wastewater, influent wastewater, river water, and riverbed sediment using the chromogenic medium mSuperCARBA (CHROMagar, MediaMage, South Africa). Prior to their identification, isolates were grown on tryptic soy agar overnight at 37°C. To identify isolated bacteria, matrix-assisted laser desorption ionization–time of flight mass spectrometry (MALDI-TOF MS) was conducted at the MALDI-TOF diagnostic service of the University of Pretoria using a MALDI Biotyper system coupled with an API 20E system (bioMérieux, Lyon, France) per the manufacturer’s protocol. Total genomic DNA was isolated and extracted using a ZymoBIOMICS DNA miniprep kit (Zymo Research, Inqaba Biotec, South Africa) per the manufacturer’s instructions. The quantity and quality of extracted DNA were analyzed using a NanoDrop 2000 spectrophotometer (Thermo Fisher Scientific, South Africa). Isolates were shipped for whole-genome sequencing at the Beijing Genomics Institute (Tai Po, Hong Kong). Prior to sequencing, the DNA quality and quantity were assessed using a Qubit double-stranded DNA (dsDNA) broad-range (BR) assay kit (Invitrogen, USA) and agarose gel electrophoresis. A standard DNA library was prepared using the Nextera XT DNA sample preparation kit (Illumina, USA). Whole-genome sequencing was carried out on a HiSeq X Ten instrument (Illumina) with paired-end read lengths of 150 bp. The raw reads were filtered to produce clean reads by removing adaptor sequences, contamination, and low-quality reads from the raw reads using AdapterRemoval v. 1.1 (3) and Sickle v. 1.33 (https://github.com/najoshi/sickle). A total of 6,321,940 (948,291,000 bp) paired-end cleaned reads were generated from the four isolates. The read quality was assessed using the FastQC tool (4). *De novo* assembly of cleaned reads was performed with SPAdes v. 3.11 (https://cge.cbs.dtu.dk/services/SPAdes/). Genome annotation was carried out on the Rapid Annotations using Subsystems Technology (RAST) v. 2.0 server1 (5). Moreover, the assembled genomes that were submitted to the *Klebsiella* multilocus sequence type (MLST) database at the Institut Pasteur (Paris, France) (http://bigsdb.pasteur.fr/klebsiella/klebsiella.html) were defined as the novel sequence type 3559 (ST3559). In addition, acquired resistance genes and virulence factors were assessed using ResFinder v. 2.1 (6) and the Virulence Factors Database (VFDB) (7), respectively. Default settings were used in all software unless otherwise noted.

The sequencing-related characteristics of the different isolated strains are presented in Table 1. All the CRKP isolates harbored antibiotic resistance genes (ARGs) conferring resistance to aminoglycosides [*aac(3)-lla*, *aac(6′)-lb-cr*, *aph(*6*)-ld*, *aph(3ʺ)-lb*, and *aadA1*], β-lactams (*bla*_OXA-48_, *bla*_CTX-M-15_, *bla*_SHV-36_, *bla*_KPC-2_, *bla*_TEM-1A_, and *bla*_OXA-1_), fluoroquinolones [*qnrS1*, *aac(6′)-lb-cr*, *oqxA*, *oqxB*, and *qnrB1*], fosfomycin (*fosA* and *fosA7*), phenicol (*catB3*), sulfonamide (*sul1* and *sul2*), tetracycline [*tet*(A)], and trimethoprim (*dfrA14* and *dfrA15*). Virulence factors found in these isolates can facilitate the pathogen to adhere, invade, and lyse targeting cells (8). These include type 3 fimbriae (*mrkA*, *mrkB*, *mrkC*, *mrkD*, *mrkF*, *mrkH*, *mrkI*, and *mrkJ*), type I fimbriae (*fimA*, *fimB*, *fimC*, *fimD*, *fimE*, *fimF*, *fimG*, *fimH*, *fimI*, and *fimK*), type IV pili (*pilW*), aerobactin (*iutA*), Ent siderophores (*entA*, *entB*, *entC*, *entD*, *entE*, *entF*, *entS*, *fepA*, *fepB*, *fepC*, *fepD*, *fepG*, and *fes*), and salmochelin (*iroE* and *iroN*).

**TABLE 1:** Characteristics and accession numbers of the isolated strains

Characteristic	Data for isolate:			
	HW1	IW3	RS3	RW2
GenBank accession no.	SAMN10992593	SAMN10992598	SAMN10992601	SAMN10992603
No. of clean reads	1,585,992	1,583,596	1,578,780	1,573,572
No. of clean bases	237,898,800	237,539,400	236,817,000	236,035,800
Fold coverage	98	94	99	84
No. of contigs	137	161	152	388
*N*_50_ (bp)	135,785	166,696	168,738	33,283
Genome length (bp)	5,602,592	5,806,598	5,575,651	5,586,912
G+C content (%)	57.1	56.7	57.2	57.2
No. of predicted coding sequences	5,579	5,857	5,555	5,742
No. of predicted RNAs	89	87	87	85
SRA accession no.	SRR8651538	SRR8651548	SRR8651547	SRR8651545

Environmental genomes are needed in order to highlight hot spots of emerging antibiotic-resistant bacteria and their spread for effective epidemiological action.

### Data availability.

The whole-genome shotgun sequencing projects of CRKP isolates HW1, IW3, RS3, and RW2 have been deposited at DDBJ/ENA/GenBank under the BioProject accession number PRJNA524761. Raw sequences have been deposited in the Sequence Read Archive (SRA) under the accession numbers listed in Table 1.
